# Survival of Hemophagocytic Syndrome Secondary to Fungal and Bacterial Infection in a Pediatric Patient with HIV: A Case Report

**DOI:** 10.3390/pathogens12081021

**Published:** 2023-08-08

**Authors:** Erika Reina-Bautista, Omar Esteban Valencia-Ledezma, María Guadalupe Frías-De-León, Gustavo Acosta-Altamirano, Carlos Alberto Castro-Fuentes

**Affiliations:** 1 Unidad de Infectología-Pediátrica, Hospital Regional de Alta Especialidad de Ixtapaluca, Carretera Federal México-Puebla Km. 34.5, Pueblo de Zoquiapan, Ixtapaluca 56530, Mexico; erika_reinab@hotmail.com; 2 Unidad de Investigación, Hospital Regional de Alta Especialidad de Ixtapaluca, Carretera Federal México-Puebla Km. 34.5, Pueblo de Zoquiapan, Ixtapaluca 56530, Mexico; 3 Unidad de Investigación Biomédica, Hospital Regional de Alta Especialidad de Ixtapaluca, Carretera Federal México-Puebla Km. 34.5, Pueblo de Zoquiapan, Ixtapaluca 56530, Mexico; 4 Posgrado en Ciencias Biológicas, Facultad de Medicina, Universidad Nacional Autónoma de México, Mexico City 04510, Mexico

**Keywords:** HLH survival, pediatric patient, AIDS, fungal infection, bacterial infection

## Abstract

HIV-associated hemophagocytic lymphohistiocytosis (HLH) is mainly due to infections caused by viruses, fungi, and, to a lesser extent, bacteria, often with fatal results. Case presentation: A 15-year-old pediatric patient from another institution was admitted to our hospital with a fever of unknown origin (FUO). Clinical analysis and laboratory studies diagnosed HIV infection. The approach to an FUO in a patient with AIDS is much more complex due to the search for common etiologies and opportunistic infections. In this case, disseminated histoplasmosis, pulmonary tuberculosis, pneumocystosis, and ehrlichiosis were diagnosed, prompting an urgent and comprehensive approach to prevent mortality. Due to the multiple infections, HLH was triggered. An early intervention with trimethoprim (TMP)–sulfamethoxazole (SMX), liposomal amphotericin B, doxycycline, and quadruple antiphimic therapy to suppress infections, in conjunction with the early administration of HLH treatment, favored the survival of this patient.

## 1. Introduction

Hemophagocytic lymphohistiocytosis (HLH) is a syndrome caused by an aggressive and potentially life-threatening immune dysregulation. It is characterized by the persistent activation of the mononuclear phagocytic system and is associated with an uncontrolled systemic hyperinflammatory response [1]. The appearance of this syndrome can be primary or secondary. Primary HLH is associated with genetic mutations that alter the cytotoxic activity of NK cells and CD8 lymphocytes. These cells accumulate in organs such as the spleen and bone marrow, where they attack erythrocytes, leukocytes, and platelets [2]. Therefore, the affliction occurs in the first years of life. Conversely, secondary HLH is triggered by infections caused by the Epstein–Barr virus (EBV), cytomegalovirus (CMV), human herpesvirus-8 (HHV-8), *Histoplasma capsulatum*, and, less frequently, the human immunodeficiency virus (HIV), as well as malignant neoplasms (lymphomas in greater frequency), autoimmune or autoinflammatory diseases, and drugs such as antiretroviral therapy (ART) [1,2,3,4].

The incidence of HIV and HLH in pediatric and non-pediatric patients is rare [1,2,3,4,5,6,7]. According to reports, the most frequently isolated infectious agents triggering HLH in HIV patients are EBV, HHV-8, *Histoplasma capsulatum*, *Mycobacterium* sp., CMV, and *Cryptococcus neoformans* [1,2,3,4,7,8]. On the other hand, some reports of ehrlichiosis caused by *Ehrlichia chaffeensis* and *E. erwingii* in non-pediatric HIV patients or non-immunosuppressed pediatric patients have been described less frequently [9].

Despite the existing literature on HIV and HLH, no concomitant infections caused by *H. capsulatum*, *P. jirovecii*, *M. tuberculosis*, and *Ehrlichia* sp. have been reported.

Therefore, in this paper, we present the case of a pediatric patient with HIV who survived HLH secondary to concomitant fungal and bacterial infections. 

## 2. Case Presentation

The pediatric patient is a 15-year-old male patient with a family history of a maternal uncle who died of AIDS 10 years ago. The patient lives in a rural area with domestic and farm animals (Atenco, State of Mexico, Mexico). He began his sexual life two months earlier with a partner of the same age. Three months before his hospital admission, he traveled to a tropical area in Mexico (Poza Rica, Veracruz, Mexico), where he worked as a loader in a vegetable warehouse. During his stay in this area, he began experiencing a fever of 38 °C and asthenia. He was seen by doctors, who prescribed several antibiotics and antipyretics without improvement, so 20 days later, he returned to his residence and presented to the Texcoco General Hospital (Hospital General de Texcoco) in the State of Mexico, Mexico, where he was hospitalized for two weeks without an etiology being determined. The patient was sent to the High Specialty Regional Hospital of Ixtapaluca (Hospital Regional de Alta Especialidad de Ixtapaluca) in the State of Mexico, Mexico, for evaluation and was admitted with a diagnosis of fever of unknown origin (FUO) using laboratories studies from another hospital. The tests included a complete blood count (CBC) that revealed a hemoglobin (Hb) level of 10.8, a hematocrit (Ht) of 31.2%, a mean corpuscular volume (MCV) of 81.8, a mean corpuscular hemoglobin (MCH) of 28.3, a platelet count of 44,000 per mm^3^, a leukocyte count of 3760 per mm^3^, a neutrophil count of 2900 per mm^3^, LT 300, and an eosinophil count of 265 per mm^3^. On the other hand, general urine test results showed a protein concentration of 30 mg/dL, a sodium (Na) value of 127, activated partial thromboplastin time (APTT) value of 37.3, the results of a liver function test, with a glutamate pyruvate transaminase (GPT) level of 241, a lactate dehydrogenase (LDH) level of 2691, a creatinine level of 0.3, and negative blood cultures. 

Upon physical examination, the patient was negative for fever, without neurological alterations, with muscle pain upon palpation, with evidence of hyperdynamic, conjunctival hyperemia with bilateral cervical lymphadenopathy <1 cm, polypnea, hypoventilation, oximetry 88%, and hepatosplenomegaly. 

During his hospitalization, the approach to the FUO revealed a positive result for HIV infection, diagnosed via serology using the ELISA method and later confirmed via Western blot. The viral load reported was 379,000 copies/mL. In addition, disease staging was performed using the CD4+ count via peripheral blood flow cytometry, with a result of 31 cells/μL (3%), corresponding to severe immunosuppression (AIDS). Virus genotyping was requested, showing HIV-1 without mutations in targeted genes. In addition, a thick drop test was performed to rule out malaria due to the history of traveling to a coastal area. The smear identified intracellular forms compatible with *Ehrlichia* sp. without serology or culture confirmation. Treatment with doxycycline was initiated, resulting in the remission of the fever, and treatment was completed in ten days. 

Other clinical findings were polypnea and desaturation, with no manifestations upon physical examination. A chest S-ray was performed which showed bilateral interstitial infiltrates, and computed axial tomography with a pulmonary window was included, reporting the following findings: “heterogeneous lung parenchyma with a diffuse reticular pattern, nodular and irregular thickening of the interlobular septa associated with the presence of ground-glass opacity, as well as bibasilar dependent opacities without bronchogram which, following the injection of the contrast medium, enhance compatibility with atelectasis”.

The pulmonology service evaluated the patient and decided to perform bronchoscopy and bronchoalveolar lavage (BAL) for pathological analysis and a PCR (polymerase chain reaction) with culture. Serum galactomannan and BAL tests were positive, as was a culture for *H. capsulatum*, which was reported nine days later. In the pathology sample, ovoid yeast-like forms were detected, some with positive budding in PAS and Grocott staining. These findings were consistent with *Histoplasma capsulatum* infection. The stain also revealed cystic shapes containing ascospores inside, confirming pneumonia due to *Pneumocystis jirovecii*. Treatment was initiated with trimethoprim–sulfamethoxazole (TMP-SMX) and steroids for respiratory compromise. In addition, fungal forms compatible with *Histoplasma* sp. were identified via bone marrow aspirate smears, and positive PCR results were reported for *M. tuberculosis* and bacilli, which were detected via auramine–rhodamine staining. Once tuberculosis and histoplasmosis were confirmed, quadruple antiphimic therapy with isoniazid, rifampicin, pyrazinamide, and ethambutol in an intensive phase at standard doses was added, as was the administration of liposomal amphotericin B at 5 mg/kg/day. 

**Figure 1:**
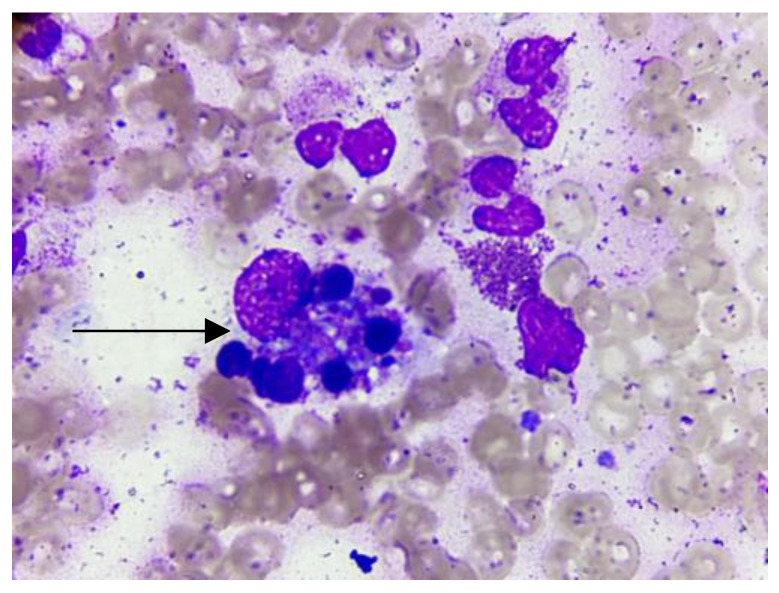
Bone marrow aspirate (arrows shows macrophages engulfing neutrophils, erythroblasts, and platelets).

The approach to an FUO for HIV patients integrated the diagnoses of secondary hemophagocytic syndrome due to the presence of persistent fever, bicytopenia (Hb 9 g/dL, platelets 60,000 per mm^3^), splenomegaly, elevated ferritin >15,000 g/μL and triglycerides 415 mg/dL, as well as hemophagocytes in the bone marrow, which were detected via the bone marrow aspirate and biopsy (Figure 1). No alterations in the cerebrospinal fluid were reported. Therefore, treatment was started with gamma globulin, 1 mg/kg/day, dexamethasone, 10 mgm2SC, cyclosporine A, and etoposide according to the HLH-2004 guideline, with the remission of the triggered infectious diseases to prevent the perpetuation of the inflammatory response. 

Other findings noted in the patient were data relating to renal failure. A biopsy was performed, reporting glomerulonephritis, collapsing variant of focal and segmental glomerulosclerosis with mesangial hyperplasia 1+, interstitial fibrosis 1+, and tubular atrophy 1+. The patient is currently being followed for stage II renal failure and treated with the steroid prescribed for pneumocystosis and HLH. It is common for AIDS patients to present euthyroid sick syndrome, especially critically ill ones. However, our patient was also diagnosed with primary hypothyroidism, exacerbated by the infection, and was treated with levothyroxine 75 mcg/kg/day. Gastroenterological, neurological, and cardiological alterations were ruled out as part of the comprehensive approach to patients with HIV (Table 1). 

**Figure 2:**
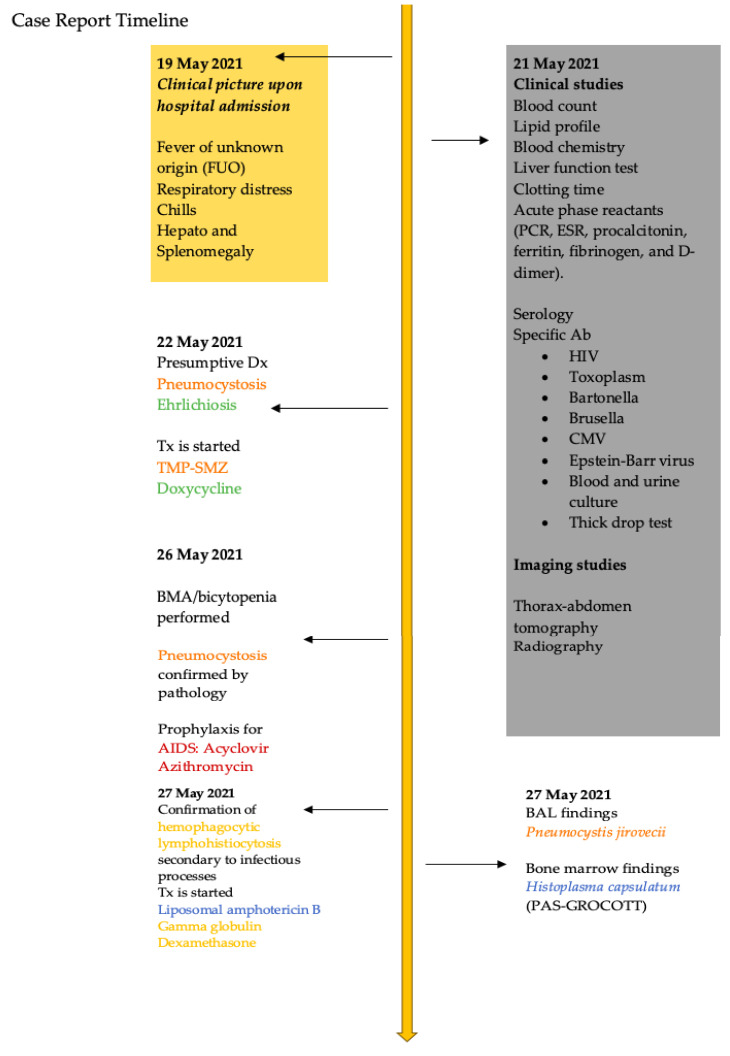

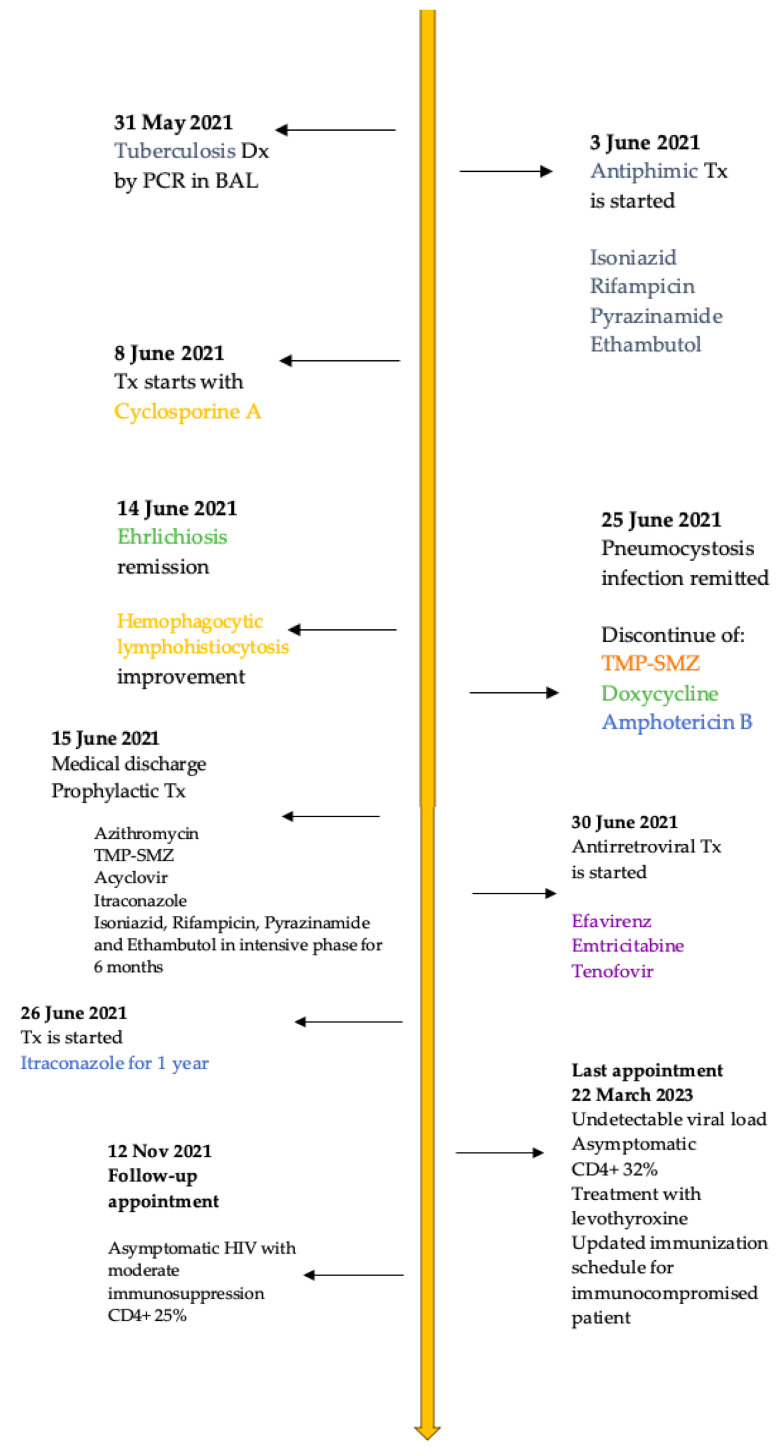

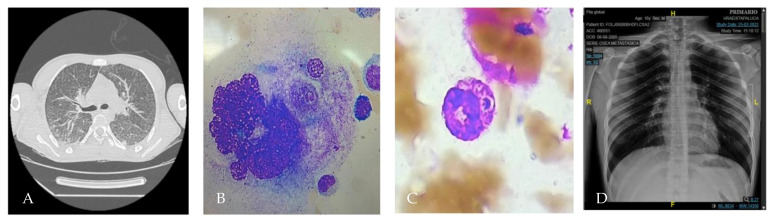
Timeline of the case, highlighting the diagnoses and treatments of the HIV-positive pediatric patient in descending chronological order (the diagnosis is highlighted in colored typography, and the corresponding treatment is indicated using the same color). (**A**) Tomography of the patient upon hospital admission showing the presence of characteristic histoplasmosis nodules; (**B**) the identification of yeasts with the typical characteristics of *Histoplasma* sp. In the bone marrow aspirate (100×); (**C**) structures of *Ehrlichia* sp. (100×); (**D**) thoracic X-ray after nine months of treatment, showing a decrease in the pulmonary radiopacity identified upon admission.

The infections were controlled within three weeks; however, remissions of histoplasmosis and tuberculosis occurred one year and six months later, respectively, following the typical duration of treatment. Once the opportunistic infections subsided, antiretroviral therapy was initiated with 600 mg of efavirenz, 246 mg of tenofovir, and 200 mg of emtricitabine orally every 24 h. The patient was discharged from the hospital with follow-up appointments at the outpatient clinic. Four months later, the patient had an undetectable viral load (CD4+ of 25%), which categorized him as not immunosuppressed (Figure 2). 

## 3. Discussion

In the present case report, we highlight the survival of HLH secondary to the fungal and bacterial infections of a pediatric patient with AIDS due to diagnostic suspicion, confirmation, and adequate and timely treatments.

At the beginning of the hospitalization, the patient presented a fever of unknown origin. The fever had been developing for over three weeks, and the patient had been hospitalized for more than three days in the hospital of origin with a fever greater than 38.3 °C without an established diagnosis. The keys to diagnosis were the anamnesis, physical examination, and laboratory studies. The first diagnosis confirmed was HIV positivity (CD4+ of 312 cells), which represented a greater diagnostic challenge since the causes of fever in an AIDS patient are much broader than in an immunocompetent individual [10]. Risk factors, such as the development of a fever in a tropical area, forced a thick drop test to rule out vector-borne infections, and ehrlichiosis was thus detected. Nonetheless, it was not possible to identify the etiological agent at the species level. Considering the clinical manifestations of pneumonia and the tomography showing different alterations, a bronchoscopy was required to facilitate the search for multiple opportunistic pathogens, finding three concomitant agents (*M. tuberculosis*, *H. capsulatum*, and *P. jirovecii*). Within the rest of the studies performed to discern the causes of the fever of unknown origin, a crucial factor was the bicytopenia that led to bone marrow aspiration. The findings showed not only fungal forms compatible with *Histoplasma* sp. but also hemophagocytosis in the bone marrow, thus completing the criteria for HLH diagnosis considering the presence of a persistent fever, pancytopenia, splenomegaly, elevated ferritin, and triglycerides. 

The literature reports the development of HLH secondary to histoplasmosis [1,7]. It is worth mentioning that in our patient, *Histoplasma* and *P. jirovecii* were identified; however, there are no reports of HLH secondary to pneumocystosis. Nonetheless, we consider that this opportunistic mycosis can cause HLH secondarily as well as *Histoplasma* sp., as the contribution of both pathogens in the fatal course of patients has been suggested [11]. The possibility of HLH presenting secondary to coinfection has been previously reported in adult patients with AIDS and diagnoses of histoplasmosis and ehrlichiosis [1,12]. Our patient was given both diagnoses. It is well known that early intervention with doxycycline can have favorable results in treating ehrlichiosis [12], like in the present work in which this treatment was introduced three days after the patient’s admission to the hospital unit. The identification of *M. tuberculosis* in our patient was probably due to the absence of ART since it limits the ability of macrophages to restrict the growth of *M. tuberculosis* and aids in immune reconstitution so that the risk of TB is reduced by up to two-thirds [13]. However, this concomitant bacterial infection has not been reported in HIV-positive patients who develop HLH. 

In patients receiving ART for HIV, the development of secondary HLH has been reported, particularly with low CD4 lymphocyte counts (26 cells/mL) [8], which is a lower level than in the present case (CD4+ 312 cells/mL). In our case, the patient was in the chronic phase of HIV infection and, unlike the case reported by Chiperi et al., the development of HLH was associated with fungal and bacterial infections before ART. 

Regarding the implementation of ART, the reports are diverse as some show favorable results [8] and others do not [14]. It is worth mentioning that despite the immunodeficiency status of the patient, he was not receiving antiretroviral therapy. However, immunomodulatory therapy (dexamethasone), etoposide, and cyclosporine A were administered according to the HLH-2004 protocol. A favorable response was observed, even in the absence of ART. Therefore, we consider the lack of antiretroviral therapy and the count of 32 cells/mL as critical factors for the survival of our patient. Immunomodulatory therapy, such as corticosteroids and intravenous immunoglobulin, represents an alternative treatment [15]. It is essential to consider that once infections are eliminated or limited as much as possible, starting antiretroviral treatment is recommended to avoid the immune reconstitution inflammatory syndrome (IRIS), as proinflammatory processes can cause death in the patient [16]. 

The diagnosed opportunistic infections were treated simultaneously according to established medical guidelines, as it is reported that patients with advanced HIV who present HLH can progress rapidly and experience a fatal disorder when an opportunistic infection is left untreated [1]. 

Reports of patients with HIV and HLH reveal that death usually occurs because of multiorgan failure due to both to the complications of the syndrome per se and to bleeding caused by thrombocytopenia and complications due to underlying diseases. In this case, our patient did not exhibit the main complications reported for this type of patient [1] except for thrombocytopenia, which occurred mildly.

## 4. Conclusions

Early intervention in patients with suspected HLH secondary to fungal or bacterial infection is crucial to avoid complications, especially when ART has not been started. In addition, it is essential to obtain a complete medical history since a patient with an FUO and AIDS can develop multiple opportunistic and non-opportunistic infections which can cause complications such as HLH.

In the present case, patient survival was associated with the use of immunomodulators (dexamethasone) and doxycycline, even when ART had not been implemented. Therefore, the efficacy of corticosteroid-based treatment for HLH stands out, parallel to the treatment of concomitant fungal and bacterial infections.

## Figures and Tables

**Table 1:** Approach to the pediatric HIV-positive patient with HLH secondary to fungal and bacterial infections.

Diagnosis	Diagnostic Methods	Treatment	Date of Diagnosis
AIDS	ELISA for positive HIV Positive Western blot CD4+ 31 cells μL/3% Viral load 379,000 copies/mL HIV-1 genotype without resistance	Efavirenz 600 mg Tenofovir 245 mg Emtricitabine 200 mg Prophylaxis for severe immunosuppression with acyclovir and azithromycin	19 May 2021 20 May 2021 26 May 2021 8 June 2021
Ehrlichiosis	Thick drop test Negative blood cultures	Doxycycline 100 mg oral every 12 h	19 May 2021
Pneumocystosis	Chest CAT scan BAL pathology report	TMP-SMX 20 mg/kg/day Dexamethasone 10 mg/m2SC day	28 May 2021
Disseminated histoplasmosis	Pathology report of BAL Findings of blastoconidia in bone marrow aspirate Positive serum galactomannan *Histoplasma capsulatum* positive culture	Amphotericin B Itraconazole	28 May 2021 29 May 2021 6 June 2021 6 June 2021
Pulmonary tuberculosis	Positive PCR in BAL Positive auramine stain in BAL Indeterminate Quantiferon Negative bacilloscopy	Isoniazid 75 mg oral every 24 h Rifampicin 150 mg oral every 24 h Pyrazinamide 300 mg oral every 24 h Ethambutol 400 mg oral every 24 h	29 May 2021 4 June 2021
Hemophagocytic lymphohistiocytosis	Fever Splenomegaly Ferritin > 15,000 μg/L Triglycerides 415 mg/dL Hemophagocytosis on bone marrow (bone marrow biopsy)	Gamma globulin 1 g/kg/day Dexamethasone Etoposide Cyclosporine A	29 May 2021

## Data Availability

Not applicable.
